# JAK-STAT pathway inhibitors in dermatology^⋆^

**DOI:** 10.1016/j.abd.2023.03.001

**Published:** 2023-05-23

**Authors:** Hélio Amante Miot, Paulo Ricardo Criado, Caio César Silva de Castro, Mayra Ianhez, Carolina Talhari, Paulo Müller Ramos

**Affiliations:** a Department of Dermatology, Faculty of Medicine, Universidade Estadual Paulista, Botucatu, SP, Brazil; b Centro Universitário Faculdade de Medicina do ABC, Santo André, SP, Brazil; c Faculdade de Ciências Médicas de Santos, Santos, SP, Brazil; d Hospital de Dermatologia Sanitária do Paraná, Curitiba, PR, Brazil; e Escola de Medicina, Pontifícia Universidade Católica do Paraná, Curitiba, PR, Brazil; f Department of Tropical Medicine and Dermatology, Instituto de Patologia Tropical e Saúde Pública, Universidade Federal de Goiás, Goiânia, GO, Brazil; g Department of Dermatology, Universidade do Estado do Amazonas, Manaus, AM, Brazil

**Keywords:** Alopecia areata, Atopic dermatitis, Janus Kinase Inhibitors, Psoriasis, Treatment, Vitiligo

## Abstract

The JAK-STAT signaling pathway mediates important cellular processes such as immune response, carcinogenesis, cell differentiation, division and death. Therefore, drugs that interfere with different JAK-STAT signaling patterns have potential indications for various medical conditions. The main dermatological targets of JAK-STAT pathway inhibitors are inflammatory or autoimmune diseases such as psoriasis, vitiligo, atopic dermatitis and alopecia areata; however, several dermatoses are under investigation to expand this list of indications. As JAK-STAT pathway inhibitors should gradually occupy a relevant space in dermatological prescriptions, this review presents the main available drugs, their immunological effects, and their pharmacological characteristics, related to clinical efficacy and safety, aiming to validate the best dermatological practice.

## Introduction

The Janus kinase (JAK) and signal transducer/activator of transcription (STAT) signaling pathway comprise a family of molecules linked to the intracellular domains of receptors for various cytokines and growth factors, which mediate their signaling to the nucleus.^1^ The naming of one of these kinases as *Janus* refers to the Roman god who represented the beginning or opening of a process, which had two faces that were associated with the two JAK domains: one catalytic and one similar to the kinase. Historically, the beginning of the day, the month and the year were intended as a sacrament to Janus, thus, according to legend, the month of January originates from his name.

The JAK-STAT pathway belongs to a complex system of protein kinases, evolutionarily conserved, in which extracellular mediators control the expression of specific genes involved in several cell functions such as mitosis, differentiation, apoptosis, hematopoiesis, development of the immune system (innate and adaptive) and exocrine gland activity. Additionally, it participates in cellular responses to insults, such as hypoxia, ultraviolet irradiation, endotoxin stimulation, oxidative and hyperosmolar stress.^2^

Thus, several inflammatory or autoimmune dermatoses are the subject of study for the indication of drugs that are JAK-STAT pathway inhibitors (JAKi).^3^ Since this therapeutic class has shown to be promising in replacing the chronic use of classic immunosuppressants (e.g., cyclosporine, azathioprine, mycophenolate, methotrexate, corticosteroids) due to its action selectivity, JAKi should gradually appear in the dermatological prescription.

This is a narrative review that included articles published on JAKi, targeting the main dermatological diseases in the MEDLINE, Scielo and Google Scholar databases, which evaluated the period from 1992 (from the first foundations of the intracellular signaling process) to the present day. The authors describe the main available drugs, their immunological effects, and their pharmacological characteristics, related to clinical efficacy and safety, aiming to support the best dermatological practice.

## The JAK-STAT pathway

**Figure 1:**
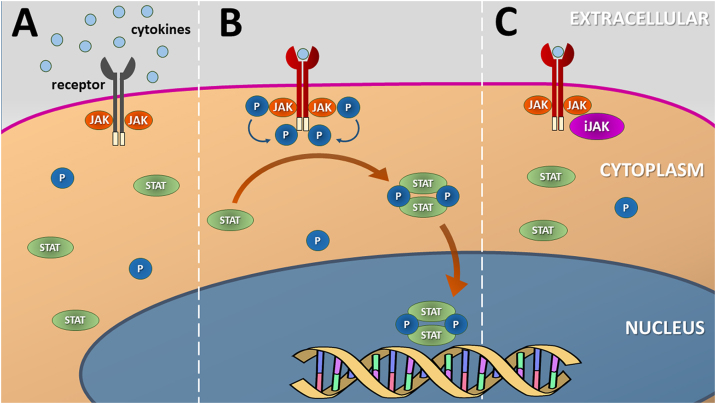
Schematic representation of the (canonical) JAK-STAT signaling pathway. (A) Several cytokines and growth factors present in the extracellular environment depend on transmembrane receptors to initiate the process of cell signaling and nuclear transcription of the genes associated with each pathway. (B) Cytokine coupling with the extracellular domain of the transmembrane receptor leads to a change in its conformation and phosphorylation of JAK dimers, located in the intracellular domain of the receptor, that transphosphorylate their terminal tyrosine residues. This process induces the dimerization and phosphorylation of inactive STAT units that migrate to the nucleus and mediate the transcription of genes related to the specific cytokine pathway. (C) Inhibitors of the JAK-STAT (JAKi) signaling pathway prevent JAK phosphorylation by disrupting cytokine or growth factor nuclear signaling. Source: the authors

JAK enzymes are tyrosine kinases attached to the intracellular domains of transmembrane receptors for certain cytokines and growth factors. After the coupling of molecules to the extracellular domains of their receptors, a conformational change occurs in their structure that leads to the phosphorylation of specific tyrosine residues in JAK dimers. This phosphorylation allows the recruitment of proteins such as STAT transcription factors, which dimerize and are translocated to the cell nucleus (via nuclear import Ran) to regulate the transcription of specific genes (Fig. 1).^4^

Each JAK subtype contains a specific tyrosine residue for ATP-dependent phosphorylation, with this specificity differentiating them among themselves, and among over 500 human tyrosine kinases.^5^

The main components of the JAK-STAT pathway are four JAK enzymes (JAK1, JAK2, JAK3 and Tyrosine Kinase 2 [Tyk2]), and seven STAT enzymes (STAT1, STAT2, STAT3, STAT4, STAT5A, STAT5B and STAT6). They are distributed as dimers, whose composition varies according to the transmembrane receptor and cell types involved.^1^ Therefore, according to the inhibition pattern for different JAK-STAT pathways, different cytokine signaling profiles are affected.

The selective blocking of cytokine groups signaling is a hallmark of the immunomodulatory effect of JAKi in comparison to conventional immunosuppressants (e.g., cyclosporine or corticosteroids), which suppress a wide range of mediators; or to immunobiological drugs, which specifically suppress one cytokine.^6^ Table 1 depicts the main cytokines and growth factors affected by blocking of JAK subtypes.

**Table 1:** Main cytokines and growth factors influenced by the signaling of different JAK subtypes in humans

JAK	Cytokines and growth factors
JAK1	IFNα, IFNβ, IFNγ, IL-2, IL-4, IL-6, IL-7, IL-9, IL-10, IL-11, IL-13, IL-15, IL-19, IL-21, IL-22, IL-27, IL-28, IL-29, IL-31, IL-35, Ciliary neurotrophic factor (CNTF), Oncostatin-M (OSM), Cardiotrophin (CT-1)
JAK2	IFNγ, IL-3, IL-5, IL-6, IL-10, IL-11, IL-13, IL-12, IL-19, IL-22, IL-23, IL-27, IL-35, granulocyte-macrophage colony stimulating factor (GM-CSF), erythropoietin (EPO), thyroid peroxidase (TPO), leptin, myeloproliferative leukemia (MPL) viral oncogene, prolactin, growth hormone (GH)
JAK3	IL-2, IL-4, IL-7, IL-9, IL-13, IL-15, IL-21
TYK2	IFNα, IFNβ, IL-10, IL-11, IL-12, IL-19, IL-22, IL-23, IL-27, IL-28, IL-29

Since JAK units are only coupled to type I and type II cytokine receptors, JAKi do not directly mediate the signaling of TNF (α, β), TGF (α, β), EGF, chemokines (e.g., IL-8, CXC, CX3C), PDGF and IL-1 (α, β), which are pathogenic in a number of dermatoses.^7^, ^8^

The JAK-STAT signaling pathway constitutes a fruitful line of research in cell biology, involving aspects related to the behavior of mutant variants of its enzymes, intracellular regulation by inhibitors/activators, effects of non-canonical activation, and interaction with other inflammatory, apoptotic, or cell growth pathways, which are medically relevant subjects but are beyond the scope of this review.^9^, ^10^, ^11^, ^12^, ^13^, ^14^, ^15^, ^16^

### Main characteristics of JAKi (in dermatology)

JAKi are small molecules (<500 kD), which favors their intestinal absorption and skin permeation, despite being little lipophilic (*log p* between 1.5 and 2). Because they are final drugs and do not depend on metabolization for the pharmacological effect, their rapid absorption leads to an early clinical effect, since their plasma peaks and elimination half-lives are also brief (<18 h).^17^

In topical formulations, JAKi do not result in high serum drug levels, minimizing systemic adverse effects and drug interactions.^17^, ^18^ Additionally, they reach high concentrations in the epidermis and upper dermis, which justifies their effectiveness in eczema, psoriasis, and vitiligo, as discussed below. However, it is recommended not to exceed 20% of the skin surface, to use a thin layer, and not to prolong its use, to reduce the chance of systemic effects.^17^

As for selectivity, JAKi can be classified as first-generation (e.g., tofacitinib, baricitinib) or non-selective; or second-generation (e.g., upadacitinib, abrocitinib, ritlecitinib) which are considered the most selective for one of the JAK subtypes. Moreover, JAKi differ in enzymatic blockade (reversible or covalent), and the binding site (type I, II, and allosteric).^19^

When considering their molecular structures, JAKi can be grouped as JAKiα (baricitinib, delgocitinib, ruxolitinib, tofacitinib) or JAKiβ (abrocitinib, upadacitinib, filgotinib, deucravacitinib). JAKiα have a structure similar to purine, and the condensed bicyclic system is formed by pyrimidine/pyrrole heterocycles. Moreover, all of these drugs exhibit a nucleophilic cyanide group for the kinase binding site. Given the similarity of the JH1 segments between the JAK isoforms, JAKiα inhibit them all, despite different affinities. JAKiβ were specifically synthesized by chemical modeling to optimize the bonds, in an attempt to combine the structure of the JAKiβ with a respective binding site, generating greater selectivity. However, JAKiβ are less comparable among themselves regarding their chemical structure.^20^

Due to the structural similarity of the JAK group enzymes, the different JAKi exert some inhibitory effect on all four of their subtypes, depending on the concentration used. Table 2 summarizes the pharmacological characteristics of the main JAKi currently used in dermatology. However, most pharmacological and pharmacodynamic indicators are estimated by assays in which inhibitory concentrations are performed by in vitro tests that employ different methodologies, without considering metabolic variations associated with sex, age group, ethnicity, body composition, comorbidities, and the concomitant use of other medications. These characteristics justify caution in their prescription and long-term follow-up, given that pharmacovigilance results of the main drugs are still in progress due to their recent approval (<5 years) by international regulatory agencies.

**Table 2:** Pharmacological characteristics of the main JAK-STAT pathway inhibitors available on the market

Drug	Inhibitory concentration (IC_50_)	Metabolism	Plasma half-life
Tofacitinib	JAK1: 2.9 nM	Hepatic CYP3A4 > CYP2C19	6 to 8h
	JAK2: 1.2 nM		
	JAK3: 1.1 nM		
	TYK2: 42 nM		
Baricitinib	JAK1: 4.0 nM	Hepatic CYP3A4	10 to 13h
	JAK2: 6.6 nM		
	JAK3: 787 nM		
	TYK2: 61 nM		
Ruxolitinib	JAK1: 6.4 nM	Hepatic CYP3A4	3 to 4h
	JAK2: 8.8 nM		
	JAK3: 487.0 nM		
	TYK2: 30.0 nM		
Ritlecitinib	JAK1: 1.638 nM	Hepatic: CYP450; Serum: glutathione-S-transferase	2 to 3h
	JAK2: 1.507 nM		
	JAK3: 0.3 nM		
	TYK2: 3.779 nM		
Abrocitinib	JAK1: 29 nM	Hepatic CYP2C19 > CYP2C9 > CYP3A4 > CYP2D6	3 to 5h
	JAK2: 803 nM		
	JAK3: > 10.000 nM		
	TYK2: 1.259 nM		
Upadacitinib	JAK1: 14 nM	Hepatic CYP3A4 > CYP2D6	6 to 15h
	JAK2: 593 nM		
	JAK3: 1.860 nM		
	TYK2: 2.715 nM		
Delgocitinib	JAK1: 2.8 nM	NA: topical use	NA: topical use
	JAK2: 2.6 nM		
	JAK3: 13.0 nM		
	TYK2: 58.0 nM		

### JAKi safety and tolerability

As with other medications, except in immunologically-mediated reactions, adverse effects (AEs) and risks associated with medications that interfere with cytokine signaling and transcription pathways, cell growth factors, and induction of cell apoptosis, are directly proportional to the following conditions: (i) Occurrence of concomitant diseases (latent tuberculosis, HIV infection, HTLV-1, Chagas disease, autoimmune diseases, inflammatory bowel disease (IBD), thrombophilia, liver, kidney and hematological diseases); (ii) Medication dosage; (iii) Treatment duration, and (iv) Metabolic pathways altered by gene polymorphism (glucose-6-phosphate dehydrogenase deficiency, slow acetylation, HLA predisposition to severe adverse reactions, as in the case of abacavir, HLA-B*5701).^21^

Nonetheless, many AEs may be infrequent, or even not present in phase III studies with a limited number of patients and short exposure to the drug, which will only be identified by pharmacovigilance reports, since most JAKi have been in the market for less than five years.^22^ The dermatologist must be aware of the possibility of AEs arising from the prolonged use of these drugs, as well as to be able to identify subgroups of susceptible patients and, mainly, the possibility of drug interactions.

### Drug interactions

Population aging and the availability of new drugs favor the concomitant use of drugs by the population, which maximizes the possibility of drug interactions, for instance, via cytochrome P450 (CYP).^23^

In patients with rheumatoid arthritis (RA) who received JAKi, it was identified that more than 10% of them were prescribed concomitant drugs with potential drug interaction, such as OAT3 (organic anion transporter 3) pump inhibitors, potent CYP3A4 and CYP2C19 inhibitors.^23^

While JAKi is metabolized in the liver by the CYP system, primarily via CYP3A4-metabolizing enzymes, the extent of the metabolism by these pathways and other enzymes varies according to the JAKi used, as well as its level of renal excretion. Table 3 provides an extensive list of drugs that potentially interfere with CYP3A4, CYP2C19 and the OAT3 pump that should be considered when using JAKi concomitantly.

**Table 3:** Medications that potentially interfere with the serum levels and safety of JAK-STAT pathway inhibitors

Increase the levels of JAKi				Reduce the levels of JAKi	
Powerful OAT3 inhibitors	Powerful CYP2C19 inhibitors	Powerful CYP3A4 inhibitors	Moderate CYP3A4 inhibitors	Weak CYP3A4 inducers	Powerful CYP3A4 inducers
Colchicine-probenecid Probenecid	Amitriptyline, Chloramphenicol, Clomipramine, Delavirdine, Fluconazole, Fluoxetine, Fluvoxamine, Gemfibrozil, Imipramine, Isoniazid, Lansoprazole, Miconazole, Stiripentol, Ticlopidine, Tioconazole, Zafirlukast	Atazanavir, Boceprevir, Clarithromycin, Cobicistat, Conivaptan, Curcumin, Danoprevir, Darunavir, Delavirdine, Econazole, Efavirenz, Elvitegravir, Ergotamine, Idelalisib, Indinavir, Itraconazole, Ketoconazole, Loperamide, Lopinavir, Mibefradil, Midostaurin, Naloxone, Nefazodone, Nelfinavir, Nilotinib, Posaconazole, Ribociclib, Ritonavir, Saquinavir, Stiripentol, Sulfamethoxazole-Trimethoprim, Telaprevir, Telithromycin, Terfenadine, Tipranavir, Troleandomycin, Voriconazole	Fusidic acid, Amiodarone, Amprenavir, Anastrozole, Aprepitant, Barnidipine, Ciclosporin, Clobazam, Clozapine, Crizotinib, Danazol, Desvenlafaxine, Diltiazem, Dimethyl sulfoxide (DMSO), Dronedarone, Erythromycin, Fluconazole, Fluvoxamine, Fosamprenavir, Fosnetupitant, Haloperidol, Imatinib, Idalpine, Isavuconazole, Isavuconazonium, Isoniazid, Isradipine, Linagliptin, Lovastatin, Luliconazole, Miconazole, Mifepristone, Milnacipran, Netupitant, Nicardapine, Nilvadipine, Paroxetine, Primaquine, Risperidone, Sertraline, Simeprevir, Tioconazole, Venetoclax, Venlafaxine, Verapamil, Zimeldine, Ziprasidone	Clonazepam, Colchicine, Diazepam, Pantoprazole, Prednisone, Ritonavir	Valproic acid, Atorvastatin, Carbamazepine, Dexamethasone, Efavirenz, Phenytoin, Phenobarbital, Rifampicin, Simvastatin

It should be noted that commonly used medications such as anxiolytic, antidepressant, antifungal, antilipidemic, and antihypertensive drugs are part of this list, and dermatologists should consider the risks of these interactions. Even recreational drugs (e.g., *Cannabis* sp.) and plant extracts (e.g., *Echinacea purpurea*, *Hypericum perforatum*), which are often omitted by patients, have an effect on the hepatic drug metabolism.^24^, ^25^ However, the brief concomitant use of JAKi with potential drugs that promote interaction, such as fluconazole for vulvovaginal candidiasis, does not imply harm.

Different JAKi for systemic use have pharmacokinetic and metabolic particularities, depending on their binding fraction with plasma proteins, rate of hepatic metabolism, and metabolite activity (Table 2).

Tofacitinib is rapidly absorbed after oral administration with peak plasma concentrations within 1 hour, reaching steady-state within 48 hours. Its oral bioavailability is 74%, regardless of food intake, and 40% of the drug is bound to plasma proteins.^20^ It is mainly eliminated through the liver (70%) and kidneys (30%), which requires lower doses in the presence of liver and kidney disease. The main route of metabolism uses CYP3A4, with a small contribution from CYP2C19, in addition to being a P-glycoprotein substrate. Interactions with midazolam, oral contraceptives, or metformin have not been demonstrated.^26^

Upadacitinib is absorbed over a wide pH range (between 1‒7.5) without major dietary interference, and only 52% is bound to plasma proteins; therefore, relevant interactions dependent on plasma protein displacement are not expected. No dose adjustment is required when upadacitinib is administered to individuals with renal impairment or those with mild or moderate liver impairment.^27^ It is metabolized by CYP3A4, with lower participation of CYP2D6. Concomitant use with ketoconazole increases Cmax (maximum drug concentration) and AUC (plasma concentration-time curve) by 70% and 75%. Concomitant use with rifampicin decreases Cmax and AUC by 50% and 60%. There is no drug interaction with warfarin, omeprazole, midazolam, and ethinylestradiol + levonorgestrel.^26^

Abrocitinib, after its absorption, binds to plasma proteins in 37%, 85% is excreted through the kidneys, and 9.5% through the stools. As for the unmetabolized form; 0.6% is excreted through the kidneys and 0.3% through the stools.^20^ Co-administration should adhere to the following recommendations: (i) Concomitant use with CYP2C19 inhibitors (abrocitinib dose should be reduced by half); (ii) Concomitant use with potent CYP2C9/CYP2C19 inducers (not recommended); (iii) OAT3 inhibitors (no need for adjustment).^28^ For examples, special attention to (i) Fluconazole, fluoxetine, fluvoxamine, and ticlopidine (CYP2C19 inhibitors); (ii) Apalutamide and rifampicin (use is not recommended), and (iii) Probenecid and teriflunomide (no dose adjustment required).^28^ Liver failure has no clinically relevant effect on the pharmacokinetics and safety of abrocitinib, thus permitting its use without dose adjustment in mild or moderate liver failure.^29^

Baricitinib is rapidly absorbed through oral administration in the fasting state, with a plasma peak within one hour, and bioavailability of 80%. High-fat and high-calorie foods reduce absorption by 29%, delaying the plasma peak to three hours. There is 50% binding to plasma proteins, and steady-state is reached within 48 hours. Less than 10% of ingested baricitinib undergoes metabolism by CYP3A4, with no metabolites being detected in the plasma, requiring dose adjustment in mild or moderate liver failure. It is excreted unchanged in urine (69%) and stools (15%), while 6% of the dosage is excreted as metabolites. Glomerular filtration is the main excretion and active secretion mechanism for OAT3, P-glycoprotein, BCRP and MATE2-K. Thus, baricitinib plasma levels increase in patients with mild (×1.4) and moderate (×2.2) renal dysfunction. In patients with creatinine clearance between 30‒60 mL/min, the dose of baricitinib should be reduced to 2 mg/d and is not recommended when creatinine clearance is <30 mL/min.^30^

Adjustment in tofacitinib or baricitinib, but not in upadacitinib dosage, is required with progression in renal failure severity. Although the dosage of tofacitinib needs to be adjusted for patients with moderate liver failure, this is not the case with baricitinib or upadacitinib.^31^ Both moderate and severe renal failure led to greater exposure to the active portion of abrocitinib, suggesting that the dosage of abrocitinib should be halved in moderate or severe renal failure.^32^

### Infections and general adverse events

The pharmacological effects of JAKi are proportional to the dosages used, so their prescription should indicate the lowest clinically effective dosage, aiming to minimize the risks associated with immunosuppression, or JAK2 inhibition, mainly hematological AEs.

The use of topical JAKi has shown few systemic AEs; however, acne, previously reported in patients receiving oral JAKi, has been observed in some reports and case series.^3^

Most infectious events related to JAKi are reported after the use of tofacitinib, since it started being marketed in 2012, reflecting the longer duration of use and the large number of patients exposed to this medication and, consequently, the greater number of publications in the literature.^33^, ^34^, ^35^, ^36^

The ORALSURV study involved 4,362 individuals with RA, older than 50 years, and with at least one cardiovascular risk factor. Patients previously receiving methotrexate were allocated to receive: tofacitinib 5 mg twice daily; tofacitinib 10 mg twice daily; or an anti-TNFα (etanercept or adalimumab). With regard to infections, the ORALSURV showed that tofacitinib resulted in a similar risk of infections as anti-TNFα, except for its propensity to reactivate latent viruses (e.g., varicella zoster, herpes simplex, and cytomegalovirus).^33^

Regarding serious infectious events (SIEs), the ORALSURV identified a similar risk for tofacitinib 5 mg and anti-TNFα use, even in patients older than 65 years.^33^ The ORALSURV data on SIEs were consistent with data from the currently approved JAKi and bDMARDs (biological disease-modifying anti-rheumatic drugs) development programs, in which the rates of SIEs were similar (3‒4 SIE/100 patient-years) with higher rates being found in the elderly.^33^, ^37^

The most common AEs reported in phase III studies of systemic JAKi were headache, nausea, and nasopharyngitis. Viral infections such as herpes simplex virus (HSV), Kaposi's varicelliform eruption (HE, herpetic eczema), and herpes zoster (HZ) are the most common dermatological AEs in AD patients receiving treatment with JAKi.^13^ Most of the data on HSV infection come from the use of tofacitinib in RA, psoriatic arthritis, and IBD. However, there is a lower incidence of HSV, HZ and HE infections in JAKi1 (selective) clinical trials in patients with AD.^38^, ^39^

The JADE REGIMEN trial enrolled 1,233 patients with moderate to severe AD in an open-label (12 weeks) phase with abrocitinib 200 mg/d as monotherapy.^39^ At the end of the 12 weeks, 798 patients (64.7%) were considered drug responders and randomly allocated to a blinded study with abrocitinib 200 mg/d, 100 mg/d, or placebo (40 weeks). Considering the open phase (induction) and blinded study, the patients completed 52 weeks of follow-up. In the induction phase (12 weeks), SIEs were observed in six patients (0.5%), with HZ occurring in nine patients (0.7%). In the blinded phase (40 weeks), the rates of SIEs and HZ varied among the patients allocated in the three different groups: (i) Placebo, 267 patients, SIEs were observed in two cases (0.7%) and HZ in two cases (0.7%); (ii) Abrocitinib 100 mg/d, 265 patients, SIEs in two cases (0.8%) and HZ in two cases (0.8%); (iii) Abrocitinib 200 mg/d, 266 patients, SIEs were observed in five patients (1.9%), and HZ in nine patients (3.4%). Adding the three groups in the blinded phase (20 weeks; 798 patients), SIEs were seen in nine cases (1.1%) and HZ in 13 patients (1.6%).^39^

In a meta-analysis on the efficacy and safety of abrocitinib, baricitinib and upadacitinib in patients with moderate to severe AD, the authors concluded that when the total number of AEs (treatment-emergent adverse events, TEAE) was analyzed, upadacitinib and abrocitinib showed greater incidence than the placebo group, and abrocitinib was associated with an increase in TEAE compared with baricitinib. However, when these JAKi were analyzed in relation to TEAE and the dosage of medication used, only the 30 mg/d dose of upadacitinib increased the incidence of TEAE, with odds ratio (OR) = 13.64. Also in the network meta-analysis, the comparison of adverse events of abrocitinib versus placebo resulted in OR = 5.57; baricitinib versus placebo, OR = 2.92; abrocitinib versus baricitinib, OR = 1.92; abrocitinib versus upadacitinib, OR = 0.41; and baricitinib versus upadacitinib, OR = 0.21. The authors of the meta-analysis point out that most clinical trial studies were conducted with efficacy and safety assessments in a short evaluation period, with no comparison yet in studies between them, and that studies of long-term efficacy and safety must be carried out.^40^

As examples of published phase III studies with a considerable sample, randomized and placebo-controlled, Measure Up 1 and Measure Up 2 evaluated 847 and 836 patients with moderate to severe AD allocated in groups receiving 30 mg/d, 15 mg/d of upadacitinib or placebo. The primary efficacy endpoints were assessed at week 16, as well as the recorded AEs (Table 4).

**Table 4:** Main adverse events, in order of frequency, in the main dermatoses where JAK-STAT pathway inhibitors are used

Dermatosis	Drug	Administration route / number of patients	Adverse events
Alopecia areata	Tofacitinib	Orally	No severe adverse events
			28.9% URTI
		90 patients^34^	one reversible leukopenia
			one elevation of transaminases that normalized with weight loss
		Orally and topical	56.8% URTI
			13.2% acne
		252 patients^35^	29.1% headache
			7.7% transaminase alteration
Vitiligo	Tofacitinib and ruxolitinib	Orally and topical	8.9% acne and transaminase alteration
		45 patients^36^	4.4% URTI
Atopic dermatitis	Baricitinib (*BREEZE AD* 1 and 2)	Orally	17.3% nasopharyngitis (1 mg dosage)
			Oral herpes simplex, headache, diarrhea
		1,239 patients^41^	One patient had thromboembolism: she was a smoker and used oral contraceptives (4 mg dose)
	Abrocitinib^a^ (*JADE MONO* 2)	Orally	14.2% nausea
			7.7% nasopharyngitis and headache
		1,233 patients^42^	5.8% acne
			5.2% vomiting
			3,2% URTI
	Upadacitinib^b^ (*Measure Up* 1)	Orally	7% to 17% acne
			9% to 13% URTI
		566 patients^43^	8% to 12% nasopharyngitis
			5% to 7% headache
			6% increased CPK

URTI, Upper respiratory tract infection; CPK, Creatine phosphokinase; Orally, Oral route.

a The dosage considered in the JADE MONO-2 study for adverse events was 200 mg/day.

b Adverse events expressed according to the dosages used (15 and 30 mg).

Regarding infectious-contagious complications, it is important to consider that disease prevalence varies according to the epidemiology of each country. The JAK-STAT pathway transmits the intracellular signaling of more than 50 cytokines and growth factors and is considered the central communication pathway of the immune system, including the defense against pathogens.

The functional alteration of the JAK-STAT system can occur not only by pharmacotherapeutic action, but also by germline loss- or gain-of-function mutations, which determine several immune phenotypes and myeloproliferative diseases. For example: (i) JAK3 loss-of-function mutations are related to severe combined immunodeficiency (SCID); (ii) Loss of function due to STAT1 mutations can generate deficiency of responses linked to interferons (IFNs), INF type 1 (alpha and beta) and INF gamma, resulting in an increase in susceptibility to viral infections; (iii) Loss-of-function mutations in JAK1, TYK2, STAT1 and STAT5B determine intracellular bacterial infections, and STATB5 deficiency can cause recurrent pneumonia; and (iv) Gain-of-function mutation in STAT1 can determine recurrent infections by *Candida* spp., as STAT1 antagonizes the antifungal effects mediated by IL-17 via STAT3, suppressing this intracellular signaling.^44^

Patients with chronic inflammatory dermatoses are at greater risk of infection due both to their disease itself (e.g., cutaneous dysbiosis with staphylococcus colonization in AD) and to the treatment they receive, particularly immunosuppressants such as cyclosporine, azathioprine, mycophenolate, and methotrexate.^44^ As an example, patients with RA have a two-fold risk of having severe infections, particularly bronchopulmonary and genitourinary ones, which contribute to higher mortality.^45^ Thus, when prescribing JAKi, one of the factors to be considered is the potential AEs of these drugs, but also the patients age (immunosenescence), associated comorbidities, and aspects of the disease pathogenesis.

Although opportunistic infections were shown to be more frequent across all classes of JAKi, no differences were seen regarding the risk of SIEs in a recent clinical trial network analysis.^45^ However, individual clinical trials have not been developed to assess rare outcomes.

An analysis of the long-term extension studies (LTEs) of tofacitinib found an incidence of SIEs of 2.7 cases per 100 patient years.^46^ When JAKi were combined with disease-modifying antirheumatic drugs (DMARDs), in a US registry of 21,832 RA patients, there was a higher occurrence of SIEs with tofacitinib associated with another DMARD at a rate of 3.67 vs. 2.01 SIEs every 100 patient-years among patients using DMARD alone. The incidence of SIE in the association of tofacitinib + DMARD was even higher than in patients who used tofacitinib + anti-TNFα: 3.67 cases/100 patient-years vs. 2.16 cases/100 patient-years.^47^, ^48^ However, these data are not yet available on dermatological conditions treated with JAKi.

Real-life data from patients with AD comparing the safety profiles of baricitinib and tofacitinib confirmed HZ as the most frequent adverse event (5.6% tofacitinib × 4.9% baricitinib).^40^ The risk is greater in the elderly, and with the co-administration of corticosteroids or methotrexate.^44^ However, there are few cases of disseminated herpes affecting several dermatomes, without evidence of visceral disease or death from herpetic infection. Clinical trials have shown that upadacitinib has a higher risk of HZ when compared to subjects using immunobiologicals and DMARDS.^44^ As with first-generation JAKi, upadacitinib in RA patients has been shown to have an increased risk of HZ when prescribed at high doses, where HZ is more likely to occur and be more severe compromising multiple dermatomes.^49^

Thus, HZ virus vaccination is an important, albeit imperfect, means of reducing the burden of HZV infection.^44^ However, both the varicella vaccine (Varivax®, for non-immune individuals under 50 years of age) and the zoster vaccine (Zostavax® for individuals ≥50 years of age) use live viruses (likewise in yellow fever vaccine) and, as such, it is recommended they are administered at least three to four weeks before starting treatment with JAKi.^44^

In Brazil, the recombinant vaccine against HZV, Shingrix®, is already available for patients over 50 years old, adults over 18 years old with immunosuppression, or those who are going to receive immunosuppressant drugs, such as JAKi. This vaccine provides protection rates of 91.3% for people aged 70 years and over, and 67.3% for immunocompromised individuals over 50 years of age. The vaccine is constituted of the viral surface glycoprotein E (gE) antigen and should be offered in two doses (0‒2 months). Since it does not use live particles, it can be given concurrently with JAKi treatment.^50^

Efficacy studies on the use of Shingrix® in an immunocompromised population aged ≥65 years and in individuals treated for IBD with immunosuppressants aged ≥50 years, showed good protection against HZ. As both (JAKi and Shingrix®) are newly available, long-term follow-up of this combination should reveal details of the immunization profile in these patients.

JAKi reduce different cytokine and growth factor receptor signaling, depending on the type of JAK inhibition. Tofacitinib, a JAK1/3 inhibitor, has important effects on the development of naïve B lymphocytes. This suggests a loss in the capacity to immunize against new antigens. A decreased immune response to pneumococcal polysaccharide vaccine 23-valent (PPSV-23) was found, especially in patients receiving concomitant methotrexate: tofacitinib, 45.1% vs. healthy controls, 68.4%. Temporary discontinuation of tofacitinib for two weeks (one week before and one week after the vaccination) did not restore the pneumococcal vaccine efficacy. The results for the influenza vaccine were not affected by the use of tofacitinib.^51^

However, most patients with psoriasis treated with tofacitinib develop adequate immunity against pneumococcal infection (pneumococcal conjugate vaccine-13) and tetanus vaccine. Vaccination with the pneumococcal conjugate vaccine-13 (PCV-13) was successful in 68% of RA patients treated with baricitinib, while only 43% achieved a ≥ 4-fold increase in antitetanic IgG concentrations. However, most patients were also taking methotrexate (89%) and/or corticosteroids (30%). Similar results were found for upadacitinib, with a satisfactory humoral response (≥2-fold increase in antibody levels) for the PCV-13 vaccine at 12 weeks in 65% and 55%, in patients using 15 mg/d and 30 mg/d.^51^

Thus, the opinion of the authors of this review is that at least three to four weeks before starting the use of systemic JAKi, the entire vaccination schedule should be updated, in accordance with the guidelines of the local health authority, including those of the Brazilian Ministry of Health. And, during treatment with JAKi, the vaccination schedule should be followed (except for live viruses), without treatment interruption.

As with other immunosuppressants and anti-TNFα immunobiologicals, there is a concern regarding *Mycobacterium tuberculosis* infection with the use of JAKi,^17^ and all patients receiving treatment with JAKi should be screened and investigated for latent or active tuberculosis.^44^ The risk of tuberculosis infection depends on the epidemiological risk of each region. The incidence of tuberculosis with tofacitinib was higher in endemic regions (0.75/100 patient-years) compared to lower-risk regions (0.02/100 patient-years).^44^, ^52^

As Brazil has endemic rates of tuberculosis, which can remain latent for decades, surveillance must occur before and during the use of immunosuppressants such as JAKi through PPD testing (Tuberculin test/Mantoux test) or interferon-gamma release assays (IGRA: ELISPOT, Quantiferon gold, Quantiferon gold plus), which show a slightly better performance.^53^, ^54^ Positive tuberculosis screening testing should be discussed with the infectologist to consider treatment of latent tuberculosis with isoniazid, for nine months, since 5% to 10% of patients show activation, especially immunosuppressed ones.

Within the scope of JAKi use, these medications bring a greater risk of general infections, risk of opportunistic infections such as *Pneumocystis jiroveci* pneumonia (PJP), HZ, tuberculosis, cytomegalovirus, and Epstein-Barr virus infections, and thus it is recommended: 1) Screening of hepatitis B virus infection prior to treatment; 2) *P. jirovecii* prophylaxis in patients with additional risk factors, such as corticosteroid use; 3) Screening for latent tuberculosis; 4) Monitoring of viral load in HBsAg negative but anti-HBcAg positive patients due to the risk of reactivation of occult hepatitis C virus infection; 5) During the chronic use of JAKi, physicians should also be aware of the increase in other opportunistic infections, such as invasive fungal infections, especially in patients who already have an additional risk, due to factors such as previous or concomitant use of corticosteroids, low blood lymphocyte count or high-dose JAKi-dependent therapy.^55^

Invasive fungal infections were detected in LTEs and in tofacitinib trials with 15 cases among 9,291 patients; *Candida* spp. was the most common, followed by cryptococcosis, histoplasmosis, and *Pneumocystis jirovecii*.^44^

### Thromboembolic events

Baricitinib, tofacitinib, ruxolitinib, and upadacitinib include warnings for potential deep vein thrombosis, pulmonary embolism, and arterial thrombosis events.^56^ Although these risks seem to be low and dosage-dependent, further studies are needed to determine the exact mechanism behind their thrombotic effects.

The finding by the ORALSURV study of an increased risk of venous thromboembolism (VTE) with a dosage of 10 mg/d of tofacitinib compared to anti-TNFα supports this fact observed for the first time in clinical trials with patients with RA at a dosage of 4 mg/d of baricitinib, reinforcing the concept that VTE can be a true adverse event related to the use of JAKi in patients with RA.^44^

Although biological plausibility is still lacking, it is reasonable to speculate that greater modulation of JAK2, observed with higher dosages of tofacitinib and baricitinib, would mediate the thrombotic effects.^44^ However, reassuringly, JAKi used in the treatment of RA at their currently approved dosages, still do not seem to carry an excessive risk of thromboembolic events.^44^

The ORALSURV study reported that tofacitinib 5 mg/d and anti-TNFα use are associated with a similar risk of VTE, and this is consistent with real-world data.^44^ Additionally, VTE incidence rates observed in the pivotal studies of tofacitinib and upadacitinib were similar (and were lower in the filgotinib studies), and the rates in the active comparator groups of these regimens (methotrexate or adalimumab) were not higher. Even for baricitinib, for which the initial imbalance in VTE risk between 2 mg/d and 4 mg/d dosages in the first 12 weeks of phase III trials raised suspicion, similar long-term incidence rates were reported for both dosages, of 0.5/100 patient-years, in line with population-based RA studies.^44^, ^57^

Finally, baricitinib 4 mg/d given for two weeks did not increase the risk of VTE in the treatment of COVID-19, a condition that carries itself an increased risk of VTE at baseline.^58^ As the ORALSURV findings suggest that there is a dosage-dependent risk with tofacitinib compared to anti-TNFα, until more substantiated research is performed with each of these compounds, it is prudent to avoid JAKi in patients with an elevated risk of VTE, particularly those with a history of VTE who are not currently anticoagulated.^44^

In 2021, the FDA issued a safety communication and required revisions of the precautionary packaging for the following JAKi: tofacitinib, baricitinib, and upadacitinib, to include information about the risk of severe cardiac events, cancer, thrombosis, and death.^55^

### Malignant neoplasms

The overall rate of occurrence of malignancies with the use of JAKi in randomized clinical trials involving RA patients and long-term extension studies was similar to that observed with bDMARDs.^33^

In the ORALSURV study, the incidence rate of malignancy (per 100 patient-years) was 1.13 for patients treated with tofacitinib at a dosage of 5 mg 2x/day and 1.13 with tofacitinib 10 mg 2x/day, in comparison with 0. 77 for anti-TNFα.^33^, ^44^

The increased risk of malignancies was driven by differential rates of several types of cancer (particularly lung cancer and lymphoma), which were seen primarily in the North-American study groups (compared to the rest of the world), among older individuals with a history of smoking.^33^ An increased risk of non-melanoma skin cancer was also observed, which was previously seen with a dosage of 10 mg/d of tofacitinib in ulcerative colitis.^59^ However, higher rates of melanoma were observed in patients using anti-TNFα, 0.09 vs. 0.02, for any dosage of tofacitinib.^59^

The mechanism by which JAKi may be associated with some types of cancer is unknown, but it is speculated that some JAKi, depending on their selectivity and effect on natural killer (NK) cells, could lower host immunosurveillance, making existing cancer or “*de novo*” have a greater possibility of evolution, as with other immunosuppressants.^60^

In summary, when oral JAKi are evaluated in general, reported rates of venous thromboembolism ranged from no events to 0.1%‒0.5% in specific phase 3 dermatology clinical trials, when compared to no events on placebo. Cardiovascular event rates ranged from no events to 0.4%‒1.2% compared to no events to 0.5%‒1.2% on placebo. Rates of severe infections were 0.4%‒4.8% compared with no events to 0.5%‒1.3% on placebo. Non-melanoma skin cancer rates ranged from no events to 0.6%‒0.9% compared with no events on placebo. Non-melanoma skin cancer rates ranged from no events to 0.2%‒0.7% compared to no events to 0.6% on placebo. Most patients who developed these adverse events had risk factors for the specific event. The most common adverse effects were upper airway infections, nausea, headache, acne, and dyslipidemia. ^61^

### Contraindications for JAKi

The main contraindication for JAKi is the uncertainty in the diagnosis of the inflammatory disease that requires treatment.^62^ Several inflammatory dermatoses can mimic cutaneous T-cell lymphoma, such as atopic dermatitis and psoriasis; alopecia can be related to syphilis; hidradenitis suppurativa, to infectious processes such as tuberculosis, actinomycosis, paracoccidioidomycosis, and metastatic Crohn's disease. The inadvertent use of JAKi in these conditions can trigger severe outcomes.

Other contraindications for JAKi include (i) Hypersensitivity to medication components (e.g., Tween 80); (ii) Tuberculosis; (iii) Altered kidney function (baricitinib); (iv) Severe liver function alteration (upadacitinib and abrocitinib); (v) Neutropenia < 500 cells/mm^3^ (baricitinib) or < 1,000 cells/mm^3^ (upadacitinib and abrocitinib); (vi) Anemia (hemoglobin ≤ 9 g/dL); (vii) Active infectious processes; (viii) Pregnant women, women wishing to become pregnant or with an active sex life and not using a contraceptive method; (ix) Thrombocytopenia < 50,000 mm^3^ (abrocitinib).^62^

The prescription should also be reconsidered if the patient shows to be unable to undergo infectious screening testing before starting treatment or regular follow-up during treatment.

### Assessment of patients before and during JAKi use

Anamnesis and general clinical examination, in addition to the dermatological examination, must be performed due to potential complications with viral, bacterial and fungal infectious diseases, in addition to neoplasms.

It is necessary to evaluate the patients history of weight loss of unknown cause, professional or family contacts with infectious diseases (tuberculosis, Chagas disease, leprosy), failure to carry out periodic cancer prevention testing indicated for the patients age group, and family history of cancer or inborn errors of immunity (immunodeficiencies).^62^

Palpation of lymph node chains, liver, spleen, pulmonary and cardiac auscultation, Giordano's sign assessment are essential in each consultation. Other data to be verified before treatment are shown in Table 5.

**Table 5:** Clinical and laboratory considerations on the use of JAK-STAT pathway inhibitors

Clinical or laboratory data	Special condition	Comment
Age range	> 65 years of age	High risk of adverse events.
	< 15 years of age	Baricitinib should not be administered.
	< 12 years of age	Do not use JAKi in children under 12 years of age.
Body weight	< 30 kg	Upadacitinib should not be used in children < 30 kg.
Lactating women		JAKi should not be used by lactating women.
Pregnancy	Absolute contraindication	Stop JAKi in individuals at risk of pregnancy.
		In case of pregnancy, discontinue use and request strict follow-up with the gynecologist/obstetrician
Hepatitis B	Positive HBsAg	Viral load ≥ 20 IU/mL (1.3 LogIU/mL): do not use JAKi
		Viral load < 20 IU/mL: monitor every 1‒3 months in addition to transaminases.
	Positive anti-HBcAg or anti-HBsAg	Verify the viral load, as in the previous situation
		If only anti-HBsAg is positive and everything else is negative (HBsAg, HBeAg, anti-HBeAg and anti-HBcAg and negative viral load), JAKi can be used.
Hepatitis C	Positive Anti-HVC	Consult a hepatologist/infectologist.
		Patients with positive anti-HCV were excluded from clinical trials of these medications.
Live virus vaccination		It should be administered 30 days before starting JAKi.
		It is contraindicated during treatment.
Active tuberculosis		The use of JAKi must be discontinued/ contraindicated.
Latent tuberculosis/ suspected asymptomatic individual	Positive PPD, IGRA/ Quantiferon gold	Tuberculosis chemoprophylaxis (isoniazid) at least three weeks before starting JAKi.
		If using JAKi: chemoprophylaxis for 6 to 9 months.
Previous history of cancer		Monitor the onset of adverse events
Renal dysfunction	Creatinine clearance > 30 mL/min/ 1.73 m^2^	Do not exceed 2 mg/d of baricitinib.
		Do not exceed 50 mg/d of abrocitinib
	Creatinine clearance ≤ 30 mL/min/ 1.73 m^2^	Upadacitinib 15 mg/d should be preferred
		Do not exceed 50 mg/d of abrocitinib
Risk of thromboembolism		Cautious use of JAKi; preference for JAK1 inhibitors.
Potentially severe infections		The use of oral JAKi is contraindicated.
Hematological alterations	Lymphopenia < 500/mm^3^	The use of oral JAKi is contraindicated.
	Neutropenia < 1.000/mm^3^	The use of upadacitinib and abrocitinib is contraindicated.
	Neutropenia < 500/mm^3^	The use of oral JAKi is contraindicated.
	Thrombocytopenia < 50.000/mm^3^	The use of abrocitinib is contraindicated.
Severe hepatic alteration	Child's Class C	The use of upadacitinib and abrocitinib is contraindicated.
Recurrent herpesvirus infection		Consider the risk of herpes reactivation with JAKi.
HIV infection		The use of oral JAKi is contraindicated.

JAKi, JAK-STAT pathway inhibitor; PPD, intradermal tuberculin test; IGRA, interferon gamma release assay.

Patients using systemic JAKi should undergo blood tests (hemogram, lipidogram, liver and kidney function), and chest X-ray, before treatment and periodically during treatment, every two months, repeating them when indicated.

### Event management during treatment with JAKi

Treatment with JAKi may promote an increase in serum lipids, with an increase in total cholesterol, LDL, HDL, and triglycerides.^62^ Cholesterol and triglyceride levels should be monitored during treatment so that, when necessary, oral antilipidemic drugs should be used according to clinical protocols.^63^

CPK elevation during treatment with JAKi is common; however, it is usually unrelated to myopathy, which requires attention to laboratory follow-up results.^62^ It is postulated that increase in CPK levels during the use of upadacitinib occurs due to JAKi1 inhibiting the physiological action of Oncostatin M (OSM), which blocks the differentiation of myoblasts into myocytes, where more CPK is produced. Once this inhibitory pathway is lost, more myoblasts differentiate into myocytes and CPK synthesis increases, without rhabdomyolysis, through cell differentiation only. It is even believed that high OSM levels determine sarcopenia in RA patients. Suspicion of rhabdomyolysis due to JAKi should include myalgia, cramps, and darkening of the urine, constituting a clinical urgency.^62^

Patients receiving JAKi should be monitored for elevated transaminases and advised to seek medical attention if they experience systemic symptoms: digestive symptoms such nausea/vomiting/anorexia and rash/pruritus/jaundice.^62^

The risk of cytopenias is greatest among patients aged > 65 years of age. JAKi should be discontinued in cases of neutropenia (< 1,000 neutrophils/mm^3^), lymphopenia (< 500 lymphocytes/mm^3^), anemia (hemoglobin < 8 g/dL), or thrombocytopenia (< 50,000 mm^3^).^62^ Neutropenia and lymphopenia increase the risk of infection and the patient should be advised to seek medical attention in case of fever, chills, sore throat or cough. If symptoms of fatigue, malaise, palpitations, dizziness or dyspnea, abdominal pain of sudden onset, nausea, vomiting, or anorexia occur, the patient must also seek medical attention due to the risk of intestinal perforation. In patients with diverticular disease of the colon, gastrointestinal perforation may follow diverticulitis; these patients should be instructed and carefully followed.^62^

Symptoms such as dry cough, dyspnea on exertion, and fever should alert the patient to seek medical attention, at risk of interstitial lung disease. In this case, blood tests should be ordered: CRP, LDH, KL-6 (Krebs von den Lungen-6), and SP-D (surfactant protein D), with the serum levels of the latter two being very sensitive in the diagnosis of drug-hypersensitivity pneumonitis.^64^ Additionally, chest radiography or computed tomography and blood gas analysis should be ordered, and JAKi should be discontinued until the diagnosis is achieved.

Pneumonia due to *P. jirovecii* can occur and the measurement of β-D glucan levels can help to establish the diagnosis. Moreover, influenza, COVID-19, *Mycoplasma*, *Chlamydia* and *Legionella* infections should be ruled out in patients with radiological images of an interstitial parenchymal pattern, which may constitute atypical non-pneumocystis pneumonia, and when infectious causes have been excluded, pneumonia of drug-induced rheumatic pulmonary disease should be considered. X-rays images showing exudate in the lung may indicate bacterial pneumonia or tuberculosis.^62^

## JAKi in inflammatory and autoimmune dermatoses

**Figure 2:**
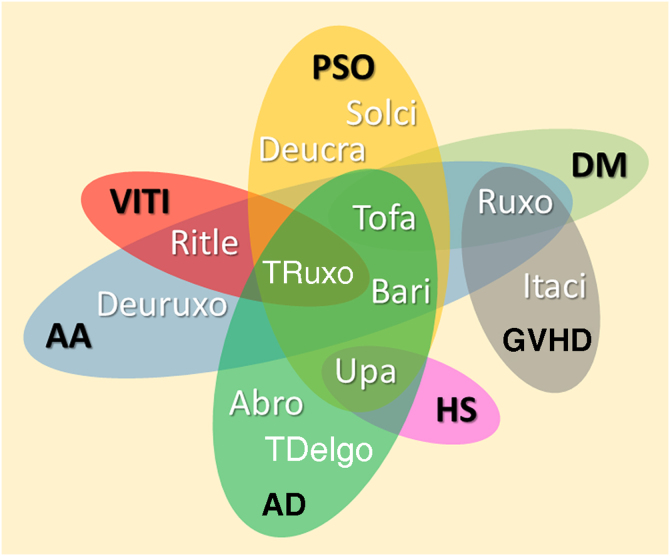
Diagram representing the main JAKi that show favorable results in clinical studies for inflammatory and autoimmune dermatoses. AA, Alopecia areata; AD, Atopic dermatitis; DM, Dermatomyositis; PSO, Psoriasis; VITI, Vitiligo; HS, Hidradenitis suppurativa; GVHD, Graft-versus-host disease; Abro, Abrocitinib; Upa, Upadacitinib; Bari, Baricitinib; TDelgo, Topical Delgocitinib; Tofa, Tofacitinib; TRuxo, Topical Ruxolitinib; Ruxo, Ruxolitinib; Ritle, Ritlecitinib; Deucra, Deucravacitinib; Solci, Solcitinib; Itaci, Itacitinib; Deuruxo, Deuruxolitinib

Most inflammatory or autoimmune dermatoses do not result from the effect of only one cytokine; however, they follow inflammatory profiles that can be inhibited by modulating the JAK-STAT pathway. The immunological basis and clinical results of the main JAKi in dermatology are shown below. Table 6 shows JAKi with dermatological indications described in the literature, and Fig. 2 summarizes the main drugs with clinical trials for use in dermatology.

**Table 6:** Dermatological indications for JAK-STAT pathway inhibitors as reported in the literature

JAKi	AD	PSO	VITI	AA	LP	LE	DM	LV	PG	GA	LN	SARC	MOR	HS	GVHD	ECZh
**Oral**																
Tofacitinib	X	X	X	X			X	X	X	X	X	X	X			
Baricitinib	X	X				X		X	X							
Abrocitinib	X															
Upadacitinib	X													X		
Ruxolitinib				X	X		X					X			X	
Delgocitinib		X														
Ritlecitinib			X	X												
Elsubritinib						X										
Itacitinib		X													X	
Peficitinib		X														
Deucravacitinib		X														
Filgotinib		X														
Solcitinib		X														
Pacritinib															X	
Deuruxolitinib				X												
Gusacitinib																X
**Topical**																
Tofacitinib	X		X	X	X					X						
Ruxolitinib	X	X	X	X							X					
Brepocitinib		X														
Delgocitinib	X															X
Cerdulatinib			X													

JAKi, JAK-STAT pathway inhibitor; AA, Alopecia areata; AD, Atopic dermatitis; DM, Dermatomyositis; PSO, Psoriasis; VITI, Vitiligo; HS, Hidradenitis suppurativa; LP, Lichen planus and Lichen planopilaris; LE, Lupus erythematosus; PG, Pyoderma gangrenosum; GA, Granuloma annulare; SARC, Sarcoidosis; NL, Necrobiosis lipoidica; LV, Livedoid Vasculopathy; MOR, Morphea; ECZh, Chronic eczema of the hands; GVHD, Graft versus host disease.

### Atopic dermatitis (AD)

AD has a high prevalence, especially in children and young adults. Its chronic and recurrent course, associated with allergic and psychological comorbidities, has an important impact on quality of life.^65^ Its pathogenesis is complex and depends on the interaction of several elements. Skin barrier failure occurs in about 80% of the patients, especially due to the filaggrin gene loss-of-function mutations. Transepidermal water loss, changes in the skin microbiome, together with antigen permeability and neural hyperreactivity favor local inflammatory response exacerbation, manifesting mainly as eczema and pruritus.^66^, ^67^

Despite being considered an inflammatory disease with a predominance of the Th2 pattern, several cytokines act together to create and maintain an environment of cutaneous hyperreactivity, which also changes in different disease stages.^3^ The involvement, especially of IL-4, IL-5, IL-10, IL-13, IL-31, IFNγ, IL-17, IL-22, and IL-33, in the cutaneous microenvironment, are indications of the potential of JAKi, (mainly JAK1) in their treatment.^67^, ^68^

Experimental models have shown that AD treatment with JAKi led to rapid pruritus suppression, skin barrier and cutaneous nerve fiber improvement, and reduction of lymphocytic infiltrate and inflammatory cytokines (e.g., IL-4 and IL-13).^69^

In Brazil, baricitinib, abrocitinib and upadacitinib are approved for oral use in moderate to severe AD; ruxolitinib 1.5% cream is available in the US market, and delgocitinib 0.5% cream should be approved in 2023. Despite being effective in the treatment of AD, no clinical trials have been developed with oral tofacitinib, and most studies are case reports and series.^67^ Table 7 summarizes pivotal clinical trial results of the main JAKi in AD.

**Table 7:** Results of main clinical studies on JAK-STAT pathway inhibitors used in the treatment of atopic dermatitis

ORAL JAKi	Study (duration)	Daily dosage	EASI75	EASI90	IGA 0/1 (or -2 points)
Upadacitinib^43^, ^69^ (> 12 years of age)	Measure Up 1 (16 weeks)	30 mg	80%	66%	62%
		15 mg	70%	53%	48%
		Placebo	16%	8%	8.4%
	Measure Up 2 (16 weeks)	30 mg	73%	62%	52%
		15 mg	60%	48%	38.8%
		Placebo	13%	8%	4.7%
	AD Up (+ topical corticoid 16 weeks)	30 mg	77%	63%	58.6%
		15 mg	65%	43%	39.6%
		Placebo	26%	13%	10.9%
Abrocitinib^42^ (> 12 years of age)	JADE MONO-1 (12 weeks)	200 mg	63%	39%	43.8%
		100 mg	40%	19%	23.7%
		Placebo	12%	5%	7.9%
	JADE MONO-2 (12 weeks)	200mg	61%	38%	38.1%
		100mg	45%	24%	28.4%
		Placebo	10%	4%	9.1%
Baricitinib^41^, ^70^ (> 18 years of age)	BREEZE AD-1 (16 weeks)	4 mg	25%	16%	17%
		2 mg	19%	11%	11%
		1 mg	17%	9%	12%
		Placebo	9%	5%	5%
	BREEZE AD-2 (16 weeks)	4 mg	21%	21%	14%
		2 mg	18%	18%	11%
		1 mg	13%	13%	9%
		Placebo	3%	6%	5%
	BREEZE AD-7 (+topical corticoid 16 weeks)	4 mg	48%	24%	31%
		2 mg	43%	17%	24%
		Placebo	23%	14%	15%

Topical JAKi	Study (duration)	Concentration 2× day	EASI 75	EASI 90	IGA 0/1 (or -2 points)
Ruxolitinib^71^	TRuE - AD 1 (8 weeks)	1.5% cream	62.1%		53.8%
		0.75% cream	56.0%		50.0%
		Vehicle	24.6%		15.1%
	TRuE - AD 2 (8 weeks)	1,5% cream	61.8%		51.3%
		0.75% creme	51.5%		39.0%
		Vehicle	14.4%		7.6%
Tofacitinib^72^	NCT02001181 (4 weeks)	2% ointment			68.0%
		Vehicle			13.0%
Delgocitinib^73^, ^74^	OBA 4-1 (4 weeks)	0.5% cream	26.4%		Body – 10.4%
					Face – 22.8%
		Vehicle	5.8%		Body ‒ 3.8%
					Face – 4.0%
	JapicCTI-184064 (4 weeks)	0.5% cream	37.7%		
		Vehicle	4.4%		

EASI75 and EASI90, ≥ 75%/ ≥ 90% reduction in eczema area and severity index; IGA 0/1, Investigator Global Assessment results in minimal erythema and noticeable papules.

In clinical studies, all systemic JAKi outperformed placebos, with rapid control of pruritus, improved quality of life and sleep, and reduced eczematous area. Higher dosages and the association of JAKi with topical corticosteroids promoted better treatment performance, albeit with a higher rate of adverse effects.

When comparing individual studies, oral abrocitinib and upadacitinib showed a greater proportion of clinical score reduction than baricitinib.^40^, ^76^ Despite differences between the studies regarding patient age groups and ethnicities, duration, and different rescue strategies, ≥ 60% of those taking upadacitinib at a dosage of 15 mg/d and ≥ 70% at a dosage of 30 mg/d are expected to achieve a ≥ 75% reduction in the Eczema Area Severity Index (EASI75).^40^, ^43^ As for abrocitinib at doses of 100 mg/d and 200 mg/d, these values are ≥ 40% and ≥ 60%. And, for baricitinib 2 mg/d and 4 mg/d, these are ≥ 18% and ≥ 21%.^40^

The 52-week extension of the AD UP study included 901 patients aged 12 to 75 years with moderate or severe AD, randomized into three groups: upadacitinib 15 mg/d, 30 mg/d and placebo (first 16 weeks), all associated with low-potency topical corticosteroid or calcineurin inhibitor 2×/d. At week 52, the proportion of patients achieving EASI75 was 50.8% for the upadacitinib 15 mg/d group and 69.0% for the 30 mg/d group, while7.4% of patients in the upadacitinib 15 mg/d group and 2.5% of the 30 mg/d group abandoned the study due to lack of efficacy.^70^

Also, in the BREEZE-AD3 study, which followed for 52 weeks 292 patients from the BREEZE-AD7 study (16 weeks), using baricitinib 2 or 4 mg/d and topical corticosteroids, a slight loss of efficacy was observed: EASI75 from 51% at week 16 to 43% at week 68. Up to 17.8% of patients treated with baricitinib 2 mg/d and 28.4% of those treated with baricitinib 4 mg/d dropped out of the follow-up due to lack of efficacy.^77^

In a real-life study that included 44 adult patients with AD treated with baricitinib, the efficacy and safety drug profile was confirmed; however, the longitudinal follow-up revealed that the percentage of patients who reached EASI75 was 53% at week four, and 33% at week 16, while 7% of the patients abandoned treatment due to insufficient efficacy.^78^ These data indicate the possibility of a slight loss of treatment efficacy from the fourth week onward.

There are still no randomized trials comparing oral JAKi with other immunosuppressants (e.g., cyclosporine, azathioprine, and methotrexate) in AD. There is also no data available regarding the association of topical and systemic JAKi, or variable dosage regimens, such as the use of high dosages in the first weeks, with subsequent dosages reduction or change in strategy to minimize costs and adverse effects.

The Heads Up study compared 692 adults with AD randomized to use oral upadacitinib 30 mg/d vs. Dupilumab 300 mg every 14 days for 24 weeks. At week 16, EASI75 was achieved by 71% of the patients using upadacitinib and 61% of those using dupilumab. The other clinical outcomes followed the superiority of upadacitinib, with emphasis on the greater and earlier reduction in pruritus. At week 24, EASI75 decreased to 64% in the upadacitinib group, while the dupilumab group resulted in 59%. Both groups required rescue treatments before week 16: upadacitinib in 20.3% and dupilumab in 17.5%.^79^

At these dosages, after 24 weeks, the Heads up study showed, in the upadacitinib group, a higher frequency of acne (18.4% vs. 3.2%), HSV (0.9% vs. 0.0%) and HZ (3.4% vs. 1.2%), whereas conjunctivitis was more frequent in the dupilumab group (10.2% vs. 1.4%). One participant from the upadacitinib group died due to bronchopneumonia associated with influenza virus infection. There was a higher frequency of laboratory alterations in the upadacitinib group. Transaminase elevation occurred in 3.4% vs. 1.1%, and two participants from the upadacitinib group abandoned the study for this reason. Anemia (2.0% vs. 0.3%), neutropenia (1.7% vs. 0.6%), and CPK elevation (7.5% vs. 3.2%) were also prevalent in the upadacitinib group.^79^ The long-term safety profile of dupilumab allows recommending its administration without the need for laboratory follow-up or risk of drug interactions.^67^, ^76^

The JADE DARE study compared abrocitinib 200 mg/d vs. dupilumab 300 mg every 14 days in 726 patients with moderate to severe AD for 26 weeks. At the end of the study, 73% of the patients in the abrocitinib group vs. 72% in the dupilumab group achieved EASI75. Conjunctivitis was more prevalent in the dupilumab group (11% vs. 3%), but the following were more frequent in the abrocitinib group: acne (13% vs. 3%), nausea (19% vs. 2%), HSV (6% vs. .5%), HZ (3% vs. 0%) and headache (13% vs. 7%).^80^

Topical JAKi (ruxolitinib and delgocitinib) confirmed the rapid effect onset (< 24 h for pruritus reduction), with a high rate of efficacy, safety and tolerability. So far, topical JAKi is indicated in cases with less than 20% of the affected body area, and non-prolonged use.^18^, ^75^, ^81^ The effectiveness of topical JAKi increases with active concentration and the number of daily applications. Moreover, the good tolerability and safety profile may prove to be appropriate in the rescue of young patients with moderate to severe AD, who often depend on topical corticosteroids for this purpose. The extended use of 1.5% ruxolitinib cream for up to 44 weeks, with 1249 adults instructed to use it on lesions if necessary, resulted in a good tolerability and safety profile, with no evidence of systemic effects. Between 74% and 78% of the patients reached the end of the study with IGA 0/1.^82^

When comparing the performances of topical JAKi in individual clinical studies, tofacitinib 2%, delgocitinib 3%, and ruxolitinib 1.5% showed greater reduction in AD scores than expected for PDE4 inhibitors (e.g., crisaborole).^78^, ^83^

In a double-blind comparison involving 51 AD patients using 0.15% triamcinolone cream 2×/d under different ruxolitinib cream concentrations and regimens for eight weeks, both triamcinolone and different dosages of ruxolitinib showed superiority over the vehicle and increase in the effect as a function of time. EASI75 occurred in 56% of patients in the group using 1.5% ruxolitinib 2×/d vs. 47% of those using triamcinolone.^84^

In general, JAKi has not yet been approved for children under 12 years, exactly the population most often afflicted by AD; however, safety studies have advanced to expand the age range of its use.

Finally, oral or topical JAKi showed to be valuable therapeutic options for AD, with a good safety profile, but their indication in AD needs to be well-defined compared to other immunosuppressants, immunobiological, and phototherapy.

### Psoriasis

The pathogenesis of psoriasis involves both the innate and adaptive immune systems, based on a complex interaction between keratinocytes, dendritic cells, T-lymphocytes, cytokines, and other inflammatory mediators. The chronic inflammation characteristic of the disease is mainly promoted by IL-23, which induces the differentiation of naïve T-cells into Th17 lymphocytes and their clonal expansion.^85^, ^86^

IL-23 signal transduction is mediated by TYK2. Activated Th17 cells release proinflammatory cytokines such as TNFα, IL-22, IL-26, and IL-29, which induce keratinocyte proliferation. In psoriasis, there are also increased serum levels of Th1 cytokines (IFNγ and IL-2), as well as decreased levels of IL-10. IL-22, produced by Th22 cells, is a cytokine-mediated by the JAK-STAT pathway and induces keratinocyte proliferation. The release of IL-22, in association with IL-15, is signaled by JAK1 and JAK3; therefore, JAK/TYK inhibition is a potential target for its treatment. However, given the pathogenesis of the disease, second-generation JAKi, selective for JAK2/TYK2, would potentially be more effective than those blocking multiple JAK/TYK isoforms.^1^, ^87^ Incidentally, individuals with genetic polymorphism and consequential loss of TYK2 function are at lower risk of developing psoriasis and other immune-mediated diseases.^88^

In general, tofacitinib (2, 5, 10 and 15 mg, 2×/d), solcitinib (200 and 400 mg, 2×/d), baricitinib (8 and 10 mg, 2×/d), and deucravacitinib (3 mg and 6 mg 2×/d) showed a PASI75 response superior to that of placebo at both week 8 and week 12 in RCTs on moderate to severe plaque psoriasis.^89^, ^90^

A meta-analysis concluded that tofacitinib (15 and 10 mg, 2×/d) and deucravacitinib (6 mg, 2×/d and 12 mg/d) had the best Physician Global Assessment (PGA) and PASI75 responses (at weeks 8 and 12) among JAKi used in the treatment of plaque psoriasis. Although the same meta-analysis showed a satisfactory safety profile of JAKi in the treatment of plaque psoriasis, tofacitinib was not approved by the FDA due to its side effects; the drug is only approved at a dosage of 5 mg 2×/d, for RA therapy. The JAKi under study for the treatment of plaque psoriasis are deucravacitinib, brepocitinib, and ropsacitinib, all TKY2 inhibitors.^89^

In Brazil, tofacitinib 5 mg 2x daily is approved for the treatment of psoriatic arthritis in adults. However, it may be effective in treating ungueal psoriasis; 33% of patients achieved NAPSI50 at week 16 on tofacitinib 5 mg 2x/d, 44% on 10 mg 2x/d, and 12% on placebo.^91^

Currently, the only topical JAKi under study for the treatment of psoriasis is brepocitinib, an orthosteric JAK1 and JAK2 inhibitor. A phase I trial, involving healthy subjects and patients with moderate to severe plaque psoriasis, confirmed its safety and good tolerability.^92^

Compared to apremilast, the only other oral drug approved for psoriasis therapy, JAK2/TYK2 inhibitors are more immunologically selective, restricting the possibility of side effects. Deucravacitinib has been approved by the FDA and by the Japanese agency for the treatment of moderate to severe plaque psoriasis in adults.^93^ A randomized study of 332 patients using 6 mg/d revealed a PASI75 response at week 16 superior to oral apremilast 30 mg 2x daily and placebo (58%; 35% and 13%). The rate of side effects was similar in the three groups. However, combining deucravacitinib with other immunosuppressants is not recommended.^94^

### Alopecia areata (AA)

AA has an autoimmune pathogenesis involving the breakdown of the immune privilege in the hair follicle. In areas of AA, an inflammatory infiltrate consisting of CD4+, CD8+, and NK lymphocytes is observed around the bulb of anagen-phase follicles, leading to disruption of melanogenesis and shaft production,^95^ with the presence of TCD8+NKG2D + lymphocytes being determinant for the development of alopecia lesions.^96^

IFNγ and IL-15 mainly induce type I cytotoxic response. CD8 + T-lymphocytes produce IFNγ, contributing to the immune privilege breakdown, inducing the production of IL-15 by epithelial cells, which in turn leads to the activation of CD8+NKGD2+ effector cells with more IFNγ production, closing the cycle that perpetuates the disease. Both the action of IFNγ on the epithelial cell and IL-15 on CD8+NKG2D + cells are mediated by the JAK-STAT pathway.^96^

Based on the report of AA regrowth with tofacitinib used in the treatment of psoriasis,^97^ multiple case series and non-comparative open-label studies have been published, showing favorable results with the use of systemic JAKi for the treatment of severe AA. The most commonly used drug was tofacitinib, followed by ruxolitinib and baricitinib.^98^, ^99^

In a comparative open study, 75 patients with AA affecting more than 30% of the scalp were randomized into two groups: tofacitinib 10 mg/d and ruxolitinib 40 mg/d. After six months of treatment, both groups showed improvement, with no difference between them. The SALT50 score was achieved by 84% of the patients in the ruxolitinib group and 78% in the tofacitinib group at six months.^100^

In two double-blind trials (BRAVE-AA1, BRAVE-AA2) patients with severe AA were randomized into three groups: baricitinib 2 mg/d, 4 mg/d or placebo. A total of 1,200 patients were enrolled for the phase III analysis of these studies at week 36. The SALT20 score was achieved by 20% and 34% in the baricitinib 2 mg/d and 4 mg/d groups, compared to 4% on placebo.^101^ The percentage of patients who discontinued treatment due to adverse effects was similar between the groups. In 2022, baricitinib received FDA approval for the treatment of AA.

In the ALLEGRO study, 718 patients were randomized into six groups: ritlecitinib 200 mg/d for four weeks and then 50 mg/d for 20 weeks; ritlecitinib 200 mg/d for four weeks and then 30 mg/d for 20 weeks; ritlecitinib 50 mg/d for 24 weeks; ritlecitinib 10 mg/d for 24 weeks, or placebo. The response to ritlecitinib was dosage-dependent, and 31% of those using 50 mg/d post-loading dosage achieved SALT20 by week 24.^102^

In a phase II trial, 149 patients were divided into four groups: deuruxolitinib 12, 8, 4 mg/d and placebo. There was a dosage-dependent response: 42%%, 26%, 14%, and 7% (placebo) of patients achieved SALT20 at week 24.^103^

A double-blind trial recruited 16 participants with universal AA who were instructed to apply 2% tofacitinib ointment, 1% ruxolitinib ointment, 0.05% clobetasol ointment, and placebo at different sites on the scalp. Six patients (38%) on tofacitinib, five (31%) on ruxolitinib, and ten (63%) on clobetasol had partial regrowth in the treatment areas, whereas none showed regrowth on placebo.^104^

In an open-label study, 1.5% ruxolitinib cream was not shown to be effective when compared to placebo,^105^ whereas 3% delgocitinib ointment also showed no SALT improvement after 12 weeks of treatment.^106^ The low efficacy of topical JAKi can be explained by the difficulty in penetrating the deep layers of the skin, which does not allow reaching the peribulbar inflammation. Vehicles that facilitate skin penetration can improve the possibilities for JAKi in AA.

Treatment of severe AA is challenging, as many patients do not respond to conventional therapy with systemic immunosuppressants or are limited by adverse effects. Severe cases have high rates of recurrence after treatment discontinuation. In a consensus organized by the Brazilian Society of Dermatology (SBD, *Sociedade Brasileira de Dermatologia*; 2020), the use of systemic JAKi is recommended as an option for extensive AA in cases that are refractory to conventional therapy.^107^ It is worth mentioning that, in the studies that allowed the approval of baricitinib by the FDA, less than 40% of the patients achieved SALT20. Additionally, patients with more than eight years of disease duration (worse prognosis) were not included. Studies comparing systemic JAKi with other immunosuppressants are essential to establish real benefits when comparing available therapies.

Maintenance of the results achieved in AA depends on treatment continuity in most cases. Longer follow-up studies are required to determine the long-term efficacy and safety of JAKi.

### Vitiligo

Vitiligo affects genetically susceptible individuals, and autoimmunity is the main cause of melanocyte aggression. Hypo/achromias, whether localized or generalized, are the manifestations of melanocyte destruction driven by cytotoxic T-lymphocytes.^108^

The two main cytokines involved in the pathogenesis of vitiligo are IFNγ and IL-15.^109^ The first is produced by tissue-resident memory T cells (TRMs) present in depigmented skin and binds to the IFNγ receptor to stimulate the expression of chemokines such as CXCL9, CXCL10 and CXCL11, which promote the infiltration of autoreactive T-cells.^110^ IL-15 is mainly produced by keratinocytes and is involved in signaling for the generation and maintenance of TRMs in damaged skin.^111^

Two JAKi are investigated for the treatment of vitiligo: 1.5% ruxolitinib cream has been approved for use in the US,^112^ and ritlecitinib 10‒50 mg/d orally is currently in a phase IIb trial.^113^ There are anecdotal reports of the use of tofacitinib 5‒10 mg/d orally and topical 2% cream or ointment in vitiligo;^114^, ^115^ however, no randomized, controlled clinical trials have been published on this drug.

The rationale for the use of JAKi in vitiligo originates from preclinical pharmacological studies that demonstrated the inhibition of the cytolytic action of CD8 + T lymphocytes and NK cells when enzymes of the kinase family (BTK, BMX, ITK, RLK and TEC) are inhibited by ritlecitinib, mainly ITK, in the case of cytotoxic T lymphocytes, and RLK and TEC in the case of NK cells. Ritlecitinib is highly selective for JAK3 and potentially inhibits IL-2 and IL-15; moreover, based on in vitro studies and cell assays, ritlecitinib may decrease the production of IFNγ released by cytotoxic T and NK cells, related to a probable ITK inhibition.^116^, ^117^

Ruxolitinib is a JAK1 and JAK2 inhibitor, whose 1.5% cream decreased the serum concentration of CXCL10, in addition to decreasing the pro-inflammatory action of chemokines and IL-15, in cell cultures of keratinocytes and melanocytes.^118^, ^119^

Tofacitinib is a JAK1 and JAK3 inhibitor. In a study with a murine model of induced vitiligo, the serum level of CXCL10 was lower in the oral tofacitinib-treated group compared with the placebo group.^120^ However, ritlecitinib has a greater JAK3 inhibitory effect when compared to tofacitinib.^117^

Two randomized, double-blind, placebo-controlled studies with 1.5% ruxolitinib cream involved patients older than 12 years with non-segmental vitiligo and an affected area of up to 10% of the body surface area. The patients were randomized to use the drug or the vehicle (2:1), twice a day, applied on the face or body for 24 weeks, after which all of them used the active substance, totaling 52 weeks. The primary endpoints were an improvement from the baseline of at least 75% of the facial VASI (Vitiligo Area Score Index), or F-VASI75 response, (F-VASI score 0 to 3) at week 24.^112^

In total, 674 patients were included, 330 in a study called TRue-V1, and 344 in the one called TRue-V2. In the first study, the percentage of patients achieving F-VASI75 response at week 24 was 29.8% in the ruxolitinib group and 7.4% in the placebo group (relative risk, 4.0; 95% confidence interval [95%CI], 1.9 to 8.4); whereas in the second study, these numbers were 30.9% and 11.4%, respectively (relative risk, 2.7; 95% CI, 1.5 to 4.9). The most common adverse effects in each study were acne (6.3 and 6.6%); nasopharyngitis (5.4 and 6.1%) and pruritus at the application site (5.4 and 5.3%), respectively.

An important secondary outcome was the 50% decrease in T-VASI (total affected area) since the start of treatment (T-VASI50). Approximately 20% of patients achieved a T-VASI50 response and 50% of patients achieved F-VASI50. This disproportion is common in vitiligo tests due to the greater concentration of follicles on the face, from where melanoblasts migrate for repigmentation.^121^, ^122^

To date, there are no randomized controlled trials with oral ruxolitinib in the treatment of vitiligo, nor topical ruxolitinib compared to or associated with the active treatments of choice: phototherapy, calcineurin inhibitors, or topical corticosteroids.

Another randomized, placebo-controlled study used dosage-escalated, oral ritlecitinib for 24 weeks, with a 24-week extension period and 8 weeks of follow-up, in patients with non-segmental vitiligo between 18 and 65 years, affected body area between 4% and 50%, and more than 25% affected facial area.^113^ The patients were randomized into five groups with ritlecitinib and one with a placebo. Two groups received a loading dosage of 100 or 200 mg/d for four weeks, followed by 50 mg/d maintenance dosage for 20 weeks. Three groups received 50, 30 or 10 mg/d for 24 weeks and one group received a placebo for 24 weeks. The patients were allocated to the extension period based on the response at week 16; non-responders (< 50% T-VASI), were allocated to an open group with brepocitinib, another open group with ritlecitinib + narrow-band UVB phototherapy (NB-UVB), or ritlecitinib 200 mg loading dosage +50 mg/d. The primary endpoint was the change from baseline in central F-VASI at week 24; the secondary endpoint was the proportion of patients with central F-VASI75 at week 24.

A total of 364 patients received treatment, and for comparison purposes with the topical ruxolitinib study, the secondary endpoint results were used. F-VASI75 at week 24 was achieved in 12.1%, 8.5%, 7.7%, 2.7%, 2.3%, and 0% in the ritlecitinib 200/50 mg, 100/50 mg, 50 mg, 30 mg, 10 mg, and in the placebo group, respectively. As for safety, 77% of the patients had some type of adverse effect, with the three most common being nasopharyngitis (15.9%), upper respiratory tract infections (11.5%), and headache (8.8%).

Despite the modest results at week 24, acceleration of repigmentation was observed after week 28. The delay in the repigmentation process is well-founded, which justifies phototherapy as an adjuvant treatment in accelerating repigmentation, at least with the anti-tyrosine kinase medications presented herein, as in the case of tofacitinib, ruxolitinib and even by the allocation of some of the non-responders with ritlecitinib to a ritlecitinib arm associated with NB-UVB.^123^, ^124^

When comparing the studies with topical ruxolitinib and oral ritlecitinib in relation to F-VASI75 at week 24, the ruxolitinib response was superior (30% vs. 12.1%).^125^ It is also relevant that this outcome was achieved with the highest dosage of ritlecitinib, the F-VASI was calculated only in the central facial area, and the ruxolitinib patients had lower phototypes than those in the ritlecitinib study, and they are more difficult to show repigmentation.^113^

To date, there are no randomized controlled trials with oral ruxolitinib in the treatment of vitiligo, nor topical ruxolitinib compared to, or associated with the topical treatments of choice: calcineurin inhibitors, or topical corticosteroids. However, in a mouse model study, either tofacitinib or ruxolitinib was administered orally, showing the superiority of tofacitinib over ruxolitinib in decreasing vitiligo scores in the affected patients.^116^, ^126^

Therefore, JAKi has shown to be potential therapeutic for vitiligo, with a good safety profile; however, this must be explored in association with other treatment measures (e.g., phototherapy) to maximize its effectiveness.

### Other dermatoses

Hidradenitis suppurative (HS) results from the dysregulation of the innate and adaptive immune system, involving several cytokines mediated by the JAK-STAT pathway (e.g., IL-17, IL-23, IL-10) and, to a lesser extent, TNFα. Tofacitinib 5 mg 2x/d has been reported in two patients with favorable results.^127^

A retrospective study with 20 patients using upadacitinib showed that, in four weeks, 75% of the individuals reached HiSCR50 and, in 12 weeks, 100%. All of them received 15 mg/d, and those who did not reach HiSCR50 within four weeks had the dosage increased to 30 mg/d.^128^ Phase II studies with INCB054707, 15 mg, 30 mg, 60 mg, and 90 mg/d demonstrated that 43%, 56%, 56%, and 88% of the patients achieved hidradenitis suppurativa clinical response (HiSCR) by week eight.^129^

Pyoderma gangrenosum (PG) is a neutrophilic inflammatory dermatosis with an estimated incidence of three to ten cases/million person-years. Its pathogenesis is poorly understood but seems to involve dysregulation of both innate and adaptive immunity.^130^ The frequency of associated immune-mediated comorbidities, and overexpression of inflammatory cytokines, in addition to the response to immunomodulatory drugs (e.g., corticosteroids, anti-TNFα, calcineurin inhibitors) support an underlying immune-mediated mechanism.^131^

There is no standardized approach to the immunosuppressive therapeutic strategy for PG, and there are resistant cases. 131, 132 Dysregulation of the JAK-STAT pathway in PG has already been demonstrated, with JAK1, JAK2, JAK3, and STAT1 to STAT6 being overexpressed in the lesions. Mutations in the JAK-STAT pathway have also been implicated in PG progression.^130^ Although there are no controlled clinical trials, several reports have demonstrated the benefits of tofacitinib, ruxolitinib, and baricitinib in PG.^132^, ^133^, ^134^, ^135^

Dermatomyositis (DM) is a multisystemic disease in which there is an activation of the innate immune system with the secretion of type I interferons. Current treatment options include a combination of photoprotection, glucocorticoids (GCs), immunosuppressants, and immunomodulators.

Tofacitinib has been successfully reported in refractory DM at doses of 5 mg 2x/d in 34 patients, 11 mg/d (extended-release) in 10 patients, and 10 mg 2x/d in two patients. Ruxolitinib was used in seven patients with a maximum dosage of up to 30 mg/d. After the start of the treatment, no recurrences were observed within three to 15 months, with improvement in skin signs, symptoms, and muscle strength tests.^136^ However, most of the studies included in this review were case series, which are susceptible to observational bias, and there was no direct comparison with another method of treatment.

Graft-versus-host disease (GVHD) develops after an allogeneic hematopoietic stem cell transplantation, when the donor T-cells not only recognize the remaining tumor cells as foreign but also the recipients tissue, leading to a potentially severe condition. Typical target organs include the skin, liver and digestive tract. Currently, all approved strategies for the treatment of GVHD are immunosuppressive therapies, such as corticoid therapy.

As GVHD is mainly characterized by increased pro-inflammatory cytokines, systemic sclerosis and damage to internal organs, inhibition of activated tyrosine kinase signaling has shown to be a promising strategy to reduce disease severity and progression. Several small molecules are being studied (phases I to III), including: ruxolitinib, itacitinib, baricitinib, ibrutinib (Bruton's tyrosine kinase inhibitor), and pacritinib.

Treatment with ruxolitinib for patients with acute GVHD refractory to corticosteroid therapy decreases patients serum levels of pro-inflammatory cytokines and the number of activated T-cells, with an overall response rate of 82%. Similar effects were observed in patients treated with itacitinib. In chronic refractory GVHD, a phase III study with ruxolitinib has shown nearly twice the achievement of primary treatment endpoints when compared to the control group.^137^

The recognition of JAKi as a therapeutic option for the treatment of chronic pruritic dermatoses started with AD. However, oral gusacitinib was recently given the “fast track” designation by the FDA for the treatment of chronic hand eczema. Moreover, clinical trials with topical delgocitinib have been initiated for chronic hand eczema in pediatric and adult patients; Moreover, as the pathogenesis of prurigo nodularis (PN) involves inflammation mediated by Th2 and Th17/Th22, which can be attenuated through JAKi, phase 2 studies are assessing the role of abrocitinib in the treatment of PN and chronic pruritus of unknown origin.^61^

## Conclusion and future research

There is an intense scientific activity linked to the development of drugs that interfere at different levels of the JAK-STAT pathway, as well as the investigation of new indications and dosage regimens.

The safety profile of JAK inhibition, especially JAK1 or JAK3, associated with the modulation of the immune response should lead to a progressive increase in the use of JAKi in comparison to immunosuppressive drugs, such as oral corticosteroids, azathioprine, mycophenolate, methotrexate, and cyclosporine, for the treatment of inflammatory and autoimmune dermatoses. However, only clinical trials with active comparators can establish JAKi regarding the best indications, dosage regimens, and pharmacoeconomic aspects.

Finally, the greater availability of molecules should lead to reduced costs for the health system and increased access to patients. On the other hand, its long-term use should clarify aspects related to the maintenance of therapeutic efficacy, the impact of the immunological blocking of selective cytokine pathways, in addition to safety regarding the emergence of infections and malignant neoplasms.

## Financial support

None declared.

## Authors' contributions

Hélio Amante Miot: Design of the study, drafting and approval of the final version of the manuscript.

Paulo Ricardo Cria: Design of the study, drafting and approval of the final version of the manuscript.

Caio César Silva de Castro: Design of the study, drafting and approval of the final version of the manuscript.

Mayra Ianhez: Design of the study, drafting and approval of the final version of the manuscript.

Carolina Talhari: Design of the study, drafting and approval of the final version of the manuscript.

Paulo Müller Ramos: Design of the study, drafting and approval of the final version of the manuscript.

## Conflicts of interest

Dr. Caio Castro: Advisory Board - Sanofi, Aché, Sun-Pharma, Galderma. Speaker - Abbvie, Jansen, Novartis, Sanofi, Leo-Pharma. Clinical Research - Abbvie, Pfizer, Jansen, Sanofi.

Dr. Paulo Criado: Clinical research for Pfizer, Novartis, Sanofi and Lilly; Advisory board Pfizer, Galderma, Takeda, Hypera, Novartis, Sanofi. Speaker: Pfizer, Abbvie, Sanofi, Hypera, Takeda, Novartis.

Dr. Hélio Miot: Advisory Board – Johnson & Johnson, L’Oréal, Theraskin and Pfizer; clinical research: Abbvie and Merz.

Dr. Mayra Ianhez: Speaker and Advisory Board - Abbvie, Sanofi-Genzyme, Pfizer, Janssen, Galderma, Theraskin.

Dr. Paulo Müller Ramos: Advisory Board – Pfizer;

Dr. Carolina Talhari: None declared.
